# Re-dosing of del Nido cardioplegia in adult cardiac surgery requiring prolonged aortic cross-clamp

**DOI:** 10.1093/icvts/ivab310

**Published:** 2021-11-11

**Authors:** Alex M D’Angelo, Samantha Nemeth, Catherine Wang, Alexander P Kossar, Koji Takeda, Hiroo Takayama, Vinayak Bapat, Yoshifumi Naka, Michael Argenziano, Craig R Smith, James Beck, Jessica Spellman, Paul Kurlansky, Isaac George

**Affiliations:** 1 Department of Surgery, Columbia University Medical Center, New York, NY, USA; 2 Center for Innovation and Outcomes Research, Columbia University, New York, NY, USA; 3 Department of Cardiothoracic Surgery, Abbott Northwestern Hospital, Minneapolis, MN, USA; 4 Department of Anesthesiology, Columbia University Medical Center, New York, NY, USA

**Keywords:** Myocardial protection, Cardioplegia, Del Nido cardioplegia

## Abstract

**OBJECTIVES:**

Few data exist on the use of del Nido cardioplegia in adults, specifically during operations requiring prolonged aortic cross-clamp. In this pilot study, we evaluate outcomes of patients undergoing surgery with cross-clamp time >3 h based on re-dosing strategy, using either full dose (FD; 1:4 blood to crystalloid ratio) or dilute (4:1 blood to crystalloid ratio) solution.

**METHODS:**

Consecutive adult patients (>18 years) undergoing cardiac surgery from 2012 to 2018 with cross-clamp time >3 h were reviewed. Patients were excluded if del Nido cardioplegia was not used. Patients were categorized into FD or dilute groups based on re-dosing solution. Propensity score matching was used to control for baseline differences between groups. The primary endpoint was in-hospital mortality. Other outcomes examined included: postoperative mechanical support, arrhythmia, stroke, dialysis and cardiac function.

**RESULTS:**

Included for analysis were 173 patients (115 male) with median age of 63.8 (interquartile range 53.9–73.1). Major comorbidities included diabetes (45), cerebrovascular disease (34), hypertension (131), atrial fibrillation (52) and previous cardiac surgery (83). There were 108 patients (62%) who received FD re-dosing, while 65 (38%) received dilute. A greater proportion of patients in the dilute group received retrograde delivery, for both induction (32/108 vs 39/65, *P *<* *0.001) and re-dose (50/108 vs 53/65, *P *<* *0.001). After propensity score matching, in-hospital mortality was not different between groups (6/48 vs 1/48, *P *=* *0.131). There were no differences in rates of postoperative mechanical circulatory support, stroke, left ventricular ejection fraction or right ventricle dysfunction.

**CONCLUSIONS:**

Del Nido cardioplegia has been used in complex cardiac surgery requiring prolonged cross-clamp. Re-dosing can be performed with either FD or dilute del Nido solution with no statistical difference in outcomes.

**Figure:**
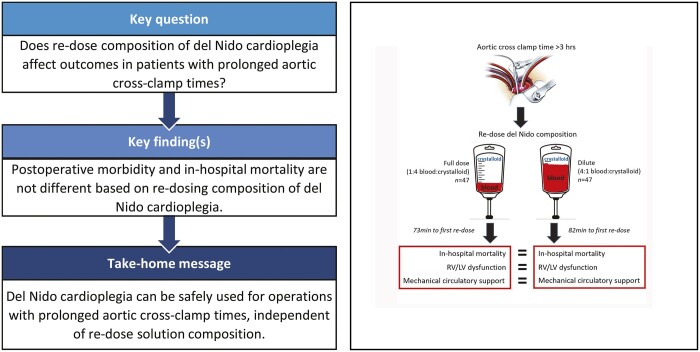

## INTRODUCTION

Since its introduction in the 1950s, there have been varying compositions and delivery techniques of cardioplegia, including both warm and cold blood, varying ratios of crystalloid and blood and a variety of chemical compositions [1]. While traditional blood-based cardioplegia can be found in various formulations, it is typically composed of a 4:1 ratio of blood to crystalloid and requires re-dosing at ∼20-min intervals after initial cardiac arrest [2–4]. Del Nido cardioplegia was developed in the early 1990s, specifically for use in the paediatric population and is administered as a single dose without the need for re-dosing up to 90 min [5, 6]. Compared to traditional blood cardioplegia, del Nido contains lidocaine, magnesium and less calcium—which serve to limit the influx of sodium into cardiac myocytes and limit calcium influx post-reperfusion. This is specifically suited to paediatric cardiac surgery as immature cardiac myocytes are especially susceptible to reperfusion injury and high levels of intracellular calcium following ischaemia [6]. Since its introduction, del Nido has been adopted for routine use in the paediatric cardiac surgical community in the USA, and more recently, has been used in the adult cardiac surgical population [7–10].

During cardiac surgery, the duration which the cross-clamp is applied has significant prognostic value for patients postoperatively, with prolonged clamp times associated with poor outcomes [11–13]. Previous trials have shown that del Nido cardioplegia resulted in significantly shorter cross-clamp times for aortic valve replacement, lower insulin requirements for both aortic and mitral valve surgery and a greater return to the spontaneous rhythm with reduced immediate postoperative inotropic support [14, 15]. For prolonged operations, cardioplegia solution must be re-dosed to ensure continued myocardial protection. The optimal re-dosing strategy for del Nido cardioplegia is unknown and frequently the result of anecdotal experience. At our institution, it has been hypothesized that dilute del Nido cardioplegia with a 4:1 blood to crystalloid ratio may be optimal for re-dosing to achieve prolonged protection of the myocardium, as this reduces the amount of crystalloid and lidocaine that a patient receives.

In this study, we sought to analyse the safety and efficacy of del Nido cardioplegia re-dosing in patients requiring aortic cross-clamp time >3 h.

## PATIENTS AND METHODS

Medical records were reviewed for adult patients undergoing cardiac surgery requiring cardioplegic arrest between 2012 and 2018 with aortic cross-clamp time >3 h. Cases were included if patients were over 18 years of age and received del Nido cardioplegia. Patients undergoing heart transplant or durable ventricular assist device implantation were excluded. Two patients were excluded due to incomplete cardioplegia dosing information. Four patients who underwent isolated coronary artery bypass graft (CABG) or adult complex congenital cardiac surgery were excluded. Patients who received a majority (>50%) of re-dose cardioplegia 1:4 blood: crystalloid were marked as ‘full dose’ (FD) and those given a majority 4:1 blood: crystalloid marked as ‘dilute’.

### Study end points

The primary endpoint was in-hospital mortality. Secondary postoperative outcomes included moderate/severe right ventricular (RV) dysfunction at the end of the operation and hospital discharge, need for postoperative mechanical circulatory support (MCS), arrhythmia, permanent pacemaker, stroke, dialysis and left ventricular (LV) function at hospital discharge.

### Data definitions

All data were obtained directly from hospital records and data definitions can be found in Supplementary Material, Table S1. Missing data are outlined in Supplementary Material, Table S2. No variable was missing more than 2%. All continuous missing preoperative data were imputed to the median and categorical data were imputed to the mode. A re-dose event was defined as cardioplegia delivery >30 min from the previous delivery. Cross-clamp time was defined by total time that aortic cross-clamp was applied, including multiple cross-clamp events during the same operation. For propensity score matching (PSM), because some patients had more than one aortic cross-clamp time, patients were categorized into a binary variable of having 1 or 2+ cross-clamps. Procedure categories were defined for propensity matching. ‘Aorta’ includes any patient who received an aortic graft, regardless of concomitant procedures. RV dysfunction was defined as moderate or severe grading based on echocardiogram. MCS included placement of intra-aortic balloon pump, veno-arterial extracorporeal membrane oxygenation and ventricular assist device.

Induction cardioplegia dosing strategy is standardized at our institution, with goal delivery of at least 1 l of FD cold del Nido cardioplegia. Delivery route is dictated by the patient’s pathology and surgeon preference. Topical cooling is not utilized at our institution. Myocardial temperature is not routinely measured. An LV vent is used on all valve/aorta cases, but not for CABG. Terminal blood cardioplegia is not used at our institution and there is no standardized reperfusion strategy for cases with prolonged aortic clamp time. As the majority of our patients underwent combined operations, Society of Thoracic Surgeons risk scores were only applicable for 25 patients (14.5%) and thus were not included in the manuscript. An alternative risk stratification score is not routinely used in these patients.

The data underlying this article will be shared on reasonable request to the corresponding author.

### Statistical analysis

*R* statistical software (version 4.0.5, R Foundation) was used for analysis and Excel (*Microsoft*) was used for figures. Data are expressed as frequencies and percentages for categorical variables. Continuous variables are expressed as either mean (SD) or median (interquartile range) depending on normality which was tested via QQ Plots and were compared using the *t*-test or Mann–Whitney test, respectively. Categorical variables were compared using Chi-square or Fisher’s exact test depending on size (>5). A *P*-value of <0.05 was deemed significant for these simple comparisons.

Logistic regression was performed with dosing strategy as the dependent variable and all preoperative risk variables in Supplementary Material, Table S3 as independent variables. Variables in the model were checked for collinearity using the variance inflation factor. No variables were found to be collinear (variance inflation factor  > 10). Next, PSM was performed with dosing strategy as the dependent variables and the same variables from Supplementary Material, Table S3 as the independent variables in the model. Patients were matched at a 1:1 ratio for normal ratio: reverse ratio and a 0.25 calliper was used. The calliper is the number of standard deviations of logit of the propensity score and used as a cut-off point in determining matches. Matching success was determined via standardized mean difference <0.1 on variables post-match. Matched groups were compared via McNemar’s test. Because all 10 outcomes involve the same dependent variable, Bonferroni correction was used to protect against inflated type 1 error. Thus, a *P*-value of 0.005 is deemed significant for this specific analysis.

### Ethical statement

This protocol (IRB-AR8359) was approved by the Columbia University Medical Center IRB with waiver of patient consent.

## RESULTS

### Cohort description

Of the 173 patients who met inclusion criteria for the study, 115 (66.5%) were male and the median age was 63.8 [53.9–73.1] years. Major comorbidities included diabetes in 45 patients (26%), cerebrovascular disease in 34 (20%), hypertension in 131 (76%) and atrial fibrillation in 52 (30%). Median preoperative left ventricular ejection fraction (LVEF) was 55% and 20 patients (11.5%) had baseline moderate/severe RV dysfunction. Previous cardiac surgery had occurred in 83 patients (48%). Coronary artery disease was present in 120 patients (69%), while 80 (46%) had moderate to severe mitral valve disease and 96 (55.5%) had moderate to severe aortic valve disease. An aortic graft was placed in 93 patients (54%). Valve only interventions were performed in 48 patients (28%) and 32 (18.5%) had combined valve/CABG. Patients in the ‘valve only’ category underwent procedures restricted to the cardiac valves, nearly all of which were multi-valve operations. Median aortic cross-clamp time for all operations was 208.0 (189.6–233.4) min and 51 patients (29.5%) had more than one cross-clamp event (Supplementary Material, Table S4).

Median induction cardioplegia was 1070.0 ml (1000.0–1330.0), with 168 patients (97%) receiving any amount of induction cardioplegia via antegrade delivery and 71 (41%) receiving any amount via retrograde delivery. Median re-dose cardioplegia was 1110.0 ml (800.0–1560.0), with 148 patients (85.5%) receiving antegrade delivery and 103 (59.5%) receiving retrograde. Median number of cardioplegia re-dose events was 3 and time to first re-dose was 74 min (59.0–91.0).

Major postoperative morbidities included: arrhythmia in 114 patients (66%), dialysis in 17 (10%), stroke in 9 (5%) and pacemaker in 34 (20%). New moderate/severe RV dysfunction developed in 33 patients (19%) by hospital discharge. Median LVEF at discharge was 55.0% (42.5–58.0). MCS was required in 38 patients (22%), with veno-arterial extracorporeal membrane oxygenation as the most common modality, with use in 17 patients (45%).

### Full dose and dilute del Nido re-dosing groups

FD del Nido re-dosing was used in 108 patients (62%), while 65 (38%) received dilute del Nido re-dosing. A greater proportion of the dilute group were male, with a history of peripheral vascular disease. Re-operative cardiac surgery occurred in 54 patients (50%) in the FD group and 29 (45%) in the dilute group (*P *=* *0.596; Table 1). Nearly all patients in the dilute group (64/65) underwent surgery from 2016 to 2018.

**Table 1:** Patient characteristics separated by re-dosing strategy

Patient characteristic	Full dose, *n* = 108, *N* (%) or median [IQR]	dilute, *n* = 65, *N* (%) or median [IQR]	*P*-value
Preoperative information			
Age (years), median [IQR]	64.4 [52.3–74.5]	62.6 [55.1–70.4]	0.604
Male, *n* (%)	60 (55.6)	55 (84.6)	<0.001
BMI, median [IQR]	27.6 [24.8–30.8]	28.7 [24.9–34.2]	0.135
Race, white, *n* (%)	62 (57.4)	43 (66.2	0.327
ESRD, *n* (%)	16 (14.8)	4 (6.2)	0.093
Pacemaker, *n* (%)	18 (16.7)	8 (12.3)	0.577
Preop. LVEF (%), median [IQR]	55.0 [50.0–60.0]	55.0 [52.5–60.0]	0.262
Preop. RV dysfunction, *n* (%)	15 (13.9)	5 (7.7)	0.326
Dialysis, *n* (%)	10 (9.3)	2 (3.1)	0.215
MI, *n* (%)	12 (11.1)	6 (9.2)	0.892
Arrhythmia, *n* (%)	40 (37.0)	27 (41.5)	0.669
Afib., *n* (%)	33 (30.6)	19 (29.2)	0.990
Chronic lung disease, *n* (%)	13 (12.0)	7 (10.8)	0.994
CVD, *n* (%)	25 (23.1)	9 (13.8)	0.196
Stroke, *n* (%)	19 (17.6)	10 (15.4)	0.868
PVD, *n* (%)	5 (4.6)	13 (20.0)	0.003
Diabetes, *n* (%)	31 (28.7)	14 (21.5)	0.389
Hypertension, *n* (%)	83 (76.9)	48 (73.8)	0.792
Endocarditis, *n* (%)	25 (23.1)	19 (29.2)	0.478
CAD, *n* (%)	83 (76.9)	37 (56.9)	0.010
Surgery status, not elective, *n* (%)	29 (26.9)	23 (35.4)	0.310
Previous cardiac surgery, *n* (%)	54 (50.0)	29 (44.6)	0.596
PCI, *n* (%)	8 (7.4)	4 (6.2)	1.00
Mitral stenosis, *n* (%)	14 (13.0)	8 (12.3)	1.00
Mitral insufficiency, *n* (%)	50 (46.3)	19 (29.2)	0.039
Aortic stenosis, *n* (%)	38 (35.2)	14 (21.5)	0.085
Aortic insufficiency, *n* (%)	33 (30.6)	23 (35.4)	0.624
Tricuspid insufficiency, *n* (%)	35 (32.4)	10 (15.4)	0.022
Hgb (g/dl), median [IQR]	12.5 [10.1–13.7]	12.9 [10.6–14.7]	0.357
WBC (×10^3^/µl), median [IQR]	7.7 [6.8–9.3]	7.7 [6.7–9.5]	0.840
Platelets (×10^3^/µl), median [IQR]	208.0 [165.0–262.0]	205.0 [163.0–254.0]	0.654
Creatinine (mg/dl), median [IQR]	1.1 [0.9–1.4]	1.1 [0.9–1.3]	0.974
Procedure, *n* (%)			<0.001
Aorta	46 (42.6)	47 (72.3)	
Valve	34 (31.5)	14 (21.5)	
Valve/CABG	28 (25.9)	4 (6.2)	
Operative/hospital information			
Cross-clamp time (min), median [IQR]	206.7 [188.3–230.6]	213.6 [189.6–240.0]	0.506
>1 cross-clamp event, *n* (%)	40 (37.0)	11 (16.9)	0.008
Total induction cardioplegia (ml), median [IQR]	1065.0 [1000.0–1312.5]	1090.0 [1000.0–1340.0]	0.794
Antegrade, *n* (%)	106 (98.1)	62 (95.4)	0.365
Retrograde, *n* (%)	32 (29.6)	39 (60.0)	<0.001
Total re-dose cardioplegia (mL), median [IQR]	1005.0 [750.0–1560.0]	1230.0 [950.0–1570.0]	0.056
Antegrade, *n* (%)	95 (88.0)	53 (81.5)	0.347
Retrograde, *n* (%)	50 (46.3)	53 (81.5)	<0.001
Total cardioplegia (ml), median [IQR]	2190.0 [1730.0–2832.5]	2500.0 [2160.0–2760.0]	0.046
Total calculated crystalloid delivered (ml), median [IQR]	1752.0 [1384.0–2266.0]	1188.0 [1046.0–1336.0]	<0.001
Time to first re-dose event (min), median [IQR]	69.5 [58.0–92.0]	76.0 [61.0–89.0]	0.915
Number of re-dose events, median [IQR]	2.0 [2.0–3.0]	3.0 [3.0–4.0]	<0.001
Intraop. blood requirement, *n* (%)	70 (64.8)	42 (64.6)	1.00
Amount received (mL), median [IQR]	1050.0 [700.0–1750.0]	1225.0 [700.0–2800.0]	0.431
Intraop. TEE LVEF (%), start of case, median [IQR]	55.0 [50.0–55.0]	55.0 [50.0–55.0]	0.520
Intraop. TEE LVEF (%), end of case, median [IQR]	50.0 [40.0–55.0]	55.0 [50.0–55.0]	0.030
New RV dysfunction, end of case TEE, *n* (%)	24 (22.2)	6 (9.2)	0.048
Postop. length of stay (days), median [IQR]	14.0 [9.0–24.0]	12.0 [7.0–23.0]	0.325

Afib: atrial fibrillation; BMI: body mass index; CABG: coronary artery bypass graft; CAD: coronary artery disease; CVD: cerebrovascular disease; ESRD: end-stage renal disease; Hgb: haemoglobin; IQR: interquartile range; LVEF: left ventricular ejection fraction; MI: myocardial infarction; PCI: percutaneous coronary intervention; PVD: peripheral vascular disease; RV: right ventricular; TEE: trans-oesophageal echocardiography; WBC: white blood cell count.

Median total cardioplegia was greater in the dilute group [2190 ml (1730.0–2832.5) vs 2500 ml (2160.0–2760.0), *P* = 0.046]. Induction cardioplegia dose did not differ between groups [1065 ml (1000.0–1312.5) vs 1090 ml (1000.0–1340.0), *P* = 0.794]. Total re-dosing cardioplegia was also not statistically different between groups [1005 ml (750.0–1560.0) vs 1230 ml (950.0–1570.0), *P* = 0.056]. A greater proportion of patients in the dilute group received retrograde cardioplegia delivery for both induction (29.6% vs 60%, *P *<* *0.001) and re-dosing (46.3% vs 81.5%, *P *<* *0.001) cardioplegia. Antegrade delivery did not differ between groups for either induction (98.1% vs 95.4%, *P *=* *0.365) or re-dosing (88.0% vs 81.5%, *P *=* *0.347). The average number of re-dose events per patient was greater in the dilute group [2.0 (2.0–3.0) vs 3.0 (3.0–4.0), *P *<* *0.001; Table 1]. The total amount of calculated crystalloid based on re-dose del Nido ratio was greater in the FD group [1752 ml (1384.0–2266.0) vs 1188 ml (1046.0–1336.0), *P* < 0.001].

Median length of stay did not differ between groups [14.0 days (9.0–24.0) vs 12.0 days (7.0–23.0), *P* = 0.325]. Median LVEF on intraoperative trans-oesophageal echocardiogram did not differ at the start of the operation, but was reduced at case conclusion [50% (40.0–55.0) vs 55% (50.0–55.0), *P *=* *0.030] in the FD group (Table 1). A greater proportion of patients in the FD group had new moderate/severe RV dysfunction at the end of the operation (22.2% vs 9.2%, *P* = 0.048).

### Propensity score matching and postoperative outcomes

As shown in Supplementary Material, Table S2, PSM resulted in 2 patient populations (48 patients each) well-matched on 11 baseline variables. Only the surgical procedure categories had a standardized mean difference >0.1. Outcomes in the propensity score matched patient groups are presented in Table 2. In-hospital mortality was not different between matched groups (12.5% vs 2.1%, *P* = 0.131). No measures of morbidity were different between groups. New moderate/severe RV dysfunction was not different between FD and dilute groups at hospital discharge (18.8% vs 12.5%, *P* = 0.606). LVEF at hospital discharge was also not different between groups [52.5% (40.0–58.5) vs 55.0% (55.0–60.0), *P* = 0.130]. Median time to first cardioplegia re-dose was also not different between groups [69.5 min (53.8–90.3) 83.0 min (60.5–89.0), *P* = 0.782].

**Table 2:** Postoperative outcomes in unmatched and propensity score matched patient groups

Patient characteristics	Unadj. full dose (*n* = 108)	Unadj. dilute (*n* = 65)	*P*-value	Adj. full dose (*n* = 48)	Adj. dilute, (*n* = 48)	*P*-value
Time to first cardioplegia re-dose (min)	69.5 [58.0–92.0]	76.0 [61.0–89.0]	0.915	69.5 [53.8–90.3]	83.0 [60.5–89.0]	0.782
MCS, *n* (%)	24 (22.2)	14 (21.5)	1.00	12 (25.0)	11 (22.9)	1.00
Arrhythmia, *n* (%)	67 (62.0)	47 (72.3)	0.225	30 (62.5)	35 (72.9)	0.404
Permanent pacemaker, *n* (%)	19 (17.6)	15 (23.1)	0.495	9 (18.8)	13 (27.1)	0.480
Stroke, *n* (%)	7 (6.5)	2 (3.1)	0.486	5 (10.4)	0 (0.0)	0.074
Dialysis, *n* (%)	13 (12.0)	4 (6.2)	0.293	5 (10.4)	1 (2.1)	0.221
Discharge LVEF, median [IQR]	53.0 [40.0–57.1]	55.0 [50.0–58.0]	0.049	52.5 [40.0–58.5]	55.0 [55.0–60.0]	0.130
New Discharge RV dysfunction, *n* (%)	26 (24.1)	7 (10.8)	0.050	9 (18.8)	6 (12.5)	0.606
In-hospital mortality, *n* (%)	16 (14.8)	4 (6.2)	0.093	6 (12.5)	1 (2.1)	0.131

IQR: interquartile range; LVEF: left ventricular ejection fraction; MCS: mechanical circulatory support; RV: right ventricular; SMD: standardized mean difference; TEE: trans-oesophageal echocardiogram.

## DISCUSSION

Despite its design specifically for use in the paediatric population, del Nido cardioplegia is increasingly utilized in the adult population, largely due to its ease of use. Del Nido requires less frequent re-dosing and has been shown to reduce aortic cross-clamp time [5, 6, 13]. A randomized controlled trial of del Nido versus cold blood cardioplegia in aortic valve replacement showed that del Nido results in less post-clamp ventricular fibrillation and no difference in mortality or morbidity [10]. In a recent meta-analysis evaluating outcomes of single-dose versus multi-dose cardioplegia strategies, del Nido cardioplegia led to reduced myocardial ischaemic time, cardiopulmonary bypass time and reperfusion fibrillation [16]. Thus, it is becoming increasingly clear that del Nido provides a safe and beneficial alternative to traditional cardioplegia options. In the current study, we investigated the use of del Nido cardioplegia in cases with a prolonged aortic cross-clamp time, focusing specifically on 2 common re-dosing strategies. We found patients re-dosed with dilute del Nido received greater total volume, with more frequent re-doses and greater retrograde delivery. However, there were no differences in morbidity or in-hospital mortality in propensity score matched groups.

The majority of studies evaluating del Nido have focused on isolated CABG or valve operations, where a single dose will suffice for the entire operation. In their large series of CABG patients, Timek *et al.* [17] routinely re-dosed del Nido at 60 min. Sanetra *et al.* [10] re-dosed at 90 min in their randomized trial of del Nido for aortic valve replacement. Animal studies lend further evidence for re-dosing intervals. Govindapillai and colleagues showed that single-dose del Nido led to superior functional recovery after 60 min cross-clamp, compared to multi-dose del Nido delivered every 20 min [18]. Nakao *et al.* [19] evaluated the effects of 90 and 120 min ischaemic time after single-dose del Nido administration in piglets. They found only minor differences in LV functional recovery when extending ischaemic time from 90 to 120 min, suggesting the safety of ischaemic time up to 120 min [19]. This is in support of the 2012 study by Charette *et al.* [5], where they proposed a re-dosing scheme for del Nido, in which a re-dose is only delivered around 90 min, if the remainder of the operation is likely to be >30 min. Effectively, the authors of that study tolerate an ischaemic time up to 2 h with a single dose of del Nido.

There is no universally agreed upon time to initially re-dose del Nido. However, an even less understood aspect of del Nido is re-dosing for extremely prolonged ischaemic times. With greater cross-clamp time and increased cardioplegia administration, fluid volume and potential solution toxicity become important points to consider. Isolated cardiac operations are declining, and combination operations have dramatically increased over the last decade—cases that require a longer cross-clamp time [20, 21]. Nearly 50% of our patient cohort were cardiac re-operations. In order to avoid theoretical electrolyte imbalances with repeated re-dosing during prolonged cases, a subset of surgeons at our institution began to re-dose del Nido in a dilute ratio (4:1 blood:crystalloid) for prolonged cases. This is supported by a report from 2006 showing that whole blood cardioplegia led to reduced myocardial oedema and increased ability to wean from cardiopulmonary bypass, compared to standard crystalloid cardioplegia [22]. The pigs in this study were dosed similar volumes of fluid, but the authors postulated that the increased crystalloid content of modified Buckberg cardioplegia led to significant oedema [22]. There are no data in the literature detailing strategies for repeated dosing of del Nido, making our study the first attempt to provide clarification.

**Figure 1:**
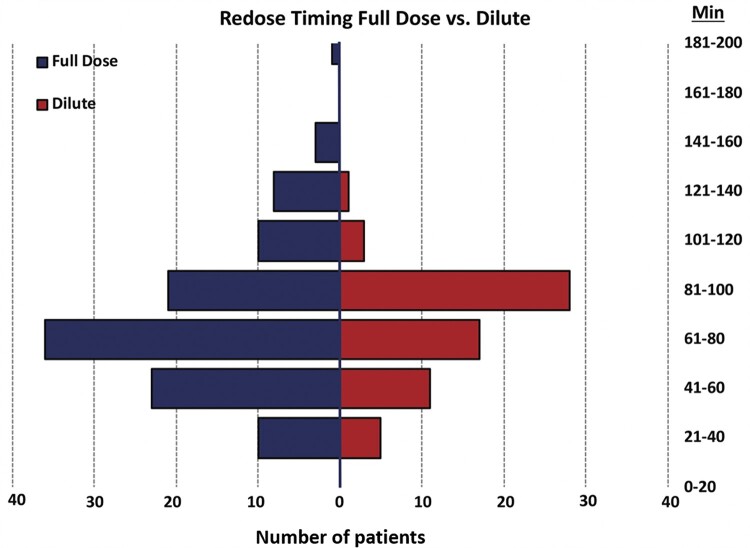
Time to first cardioplegia re-dose based on group, with a median time of 70–80 min in both full dose and dilute groups.

**Figure 2:**
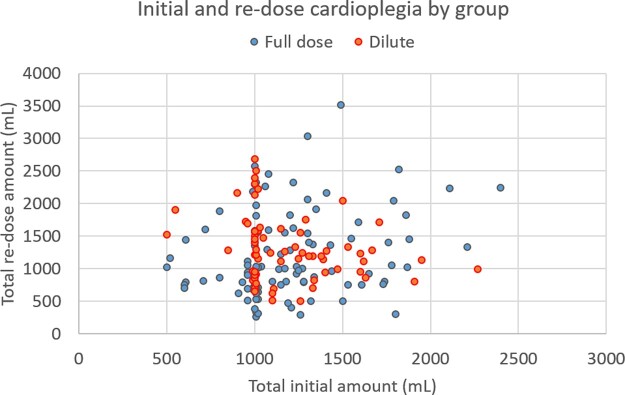
Total induction and total re-dose cardioplegia volume by group, with no statistical difference in induction cardioplegia amount, but greater re-dose cardioplegia delivered in the dilute group (*P *=* *0.044).

In our study, the median time to first re-dose was between 65 and 80 min after initial dose for both groups (Fig. 1), which is congruent with the above-described reports. Patients receiving dilute del Nido were significantly more likely to have retrograde delivery, both with induction and re-dosing cardioplegia, which is likely a result of specific pathology and surgeon preference. Ultimately, total induction cardioplegia was not different between groups (Fig. 2). Total re-dose cardioplegia was also not different between groups, albeit with a borderline *P*-value (0.056). However, total overall cardioplegia was significantly greater in the dilute group). The aetiology of this finding is unclear, but it is possible the surgeons felt it necessary to slightly increase volume of cardioplegia to compensate for the dilute ratio. Moreover, the total volume of crystalloid may have more impact on clinical outcomes—as expected, the volume of total crystalloid given in the dilute group was nearly 1.5 times lower than the FD group. However, any such association between volume of crystalloid administration and outcome is speculative. After PSM, there were no differences in morbidity or in-hospital mortality based on re-dosing strategy. Finally, there was no evidence of adverse events from del Nido administration in higher doses or volumes, such as refractory myocyte inactivity or lidocaine toxicity.

What is apparent from our cohort is the significant morbidity and mortality in this patient population. The in-hospital mortality rate for all patients was >11%, with MCS use in more than 20% of patients. Reported mortality from a Society of Thoracic Surgeons database review of cases with cross-clamp time >5 h, showed a 30-day mortality of 12.4% [23]. While there were no significant differences in outcome after PSM in our study, there were a few notable disparities between groups. The in-hospital mortality rate in FD patients was nearly six times that of dilute patients (12.5% vs 2.1%) and the incidence of moderate to severe RV dysfunction at hospital discharge in the FD group was greater than in the dilute group (18.8% vs 12.5%). Discharge LV function was also slightly lower in the FD cohort. In addition, the rate of stroke (10.4% vs 0%) and new dialysis requirement (10.4% vs 2.1%) were higher in the FD group. These disparities did not reach statistical significance, but could be indicative of underlying differences that were unable to be distinguished with our relatively small sample size and heterogeneous cohort, as each matched group consisted of only 48 patients. Alternatively, this could signal improved protection with a higher volume, lower crystalloid, blood-based cardioplegia strategy for prolonged cross-clamp cases, with increased utilization of retrograde delivery. Regardless, careful adherence to surgical principles is warranted, and special care must be given to right coronary protection, especially when retrograde cardioplegia is utilized.

Given these findings, it appears FD and dilute del Nido cardioplegia are both reasonable options for re-dosing during operations with prolonged aortic cross-clamp time, where multiple re-doses are required. A larger patient sample would be required to detect subtle differences between groups, which did not reach statistical significance in our cohort. Ultimately, we recommend induction dosing with 1 l of FD del Nido and initial re-dose 60–90 min later with either FD or dilute solution if cross-clamp time is expected to be <3 h, and dilute re-dosing for cross-clamp over 3 h. If particular volume or electrolyte concerns are present, this may guide choice of re-dosing agent as appropriate.

### Study limitations

Cardioplegia is difficult to study due to variability in composition, dosing and dosage timing and there are several limitations to our study. Given the extremely heterogeneous nature of operations within our cohort, we were unable to propensity match for specific procedures. We were, however, able to match for broad procedure category. We were also unable to match for individual surgeon, as specific surgeons at our institution are more likely to re-dose with dilute del Nido. Patients with multiple cross-clamp events were included in the analysis based on total clamp time. However, groups were propensity matched to ensure equal distribution of multi-clamp patients, limiting its potential effect on results. A further limitation was the necessity to categorize patients for re-dose cardioplegia type as a binary variable. While most patients in each category received nearly 100% of the designated re-dose formulation, a minority of patients received a mix and were categorized by majority of re-dose agent. We do not routinely collect comprehensive postoperative laboratory values and tests such as troponin and amylase are not available. There is potential calendar time bias, as nearly all patients in the dilute group underwent surgery in the latter half of the study period. Finally, this study represents the clinical experience at a single institution examined in a retrospective manner and the findings may not be uniformly transferrable to other centres. As with all retrospective studies, there is possible selection bias and inability to infer causality.

## CONCLUSIONS

We have reported outcomes in the first study to date of various re-dosing strategies of del Nido cardioplegia in operations with prolonged aortic cross-clamp time. Del Nido cardioplegia is becoming widely utilized in the adult population, with minimal data on re-dosing methods for prolonged cases. In this study, we report no statistical difference in outcomes with FD and dilute del Nido as a re-dose solution at 65–80 min after initial dose. It is clear that further investigation is needed to delineate optimal re-dosing methods, but this report brings to attention the initial success of multiple strategies.

## SUPPLEMENTARY MATERIAL

Supplementary material is available at *ICVTS* online.

**Conflict of interest:** none declared. 

## Author contributions

**Alex M. D'Angelo:** Conceptualization; Data curation; Formal analysis; Methodology; Writing—original draft. **Samantha Nemeth:** Formal analysis; Methodology. **Catherine Wang:** Conceptualization; Data curation; Investigation; Methodology. **Alexander P. Kossar:** Conceptualization; Investigation; Methodology; Project administration. **Koji Takeda:** Methodology; Project administration; Writing—review & editing. **Hiroo Takayama:** Methodology; Supervision; Writing—review & editing. **Vinayak Bapat:** Investigation; Methodology. **Yoshifumi Naka:** Conceptualization; Methodology; Writing—review & editing. **Michael Argenziano:** Conceptualization; Methodology. **Craig R. Smith:** Conceptualization; Methodology. **James Beck:** Data curation; Investigation; Methodology. **Jessica Spellman:** Conceptualization; Data curation; Methodology; Project administration. **Paul Kurlansky:** Conceptualization; Data curation; Formal analysis; Methodology. **Isaac George:** Conceptualization; Investigation; Methodology; Resources; Supervision; Writing—review & editing.

## Reviewer information

Interactive CardioVascular and Thoracic Surgery thanks the other anonymous reviewers for their contribution to the peer review process of this article.

## Supplementary Material

ivab310_Supplementary_Data

## ABBREVIATIONS

CABG: Coronary artery bypass graft
FD: Full dose
LV: Left ventricular
LVEF: Left ventricular ejection fraction
MCS: Mechanical circulatory support
PSM: Propensity score matching
RV: Right ventricular
