# Supplementary material for: Re-dosing of del Nido cardioplegia in adult cardiac surgery requiring prolonged aortic cross-clamp
Source: Interact Cardiovasc Thorac Surg. 2021 Nov 11;34(4):556–63. doi: 10.1093/icvts/ivab310 (PMC8972223; doi:10.1093/icvts/ivab310)
Supplement: ivab310_Supplementary_Data [file ivab310_supplementary_data.docx]

Supplementary Table 1 – Data definitions

| **Data Point** | **Definition** |
| --- | --- |
| Race | race documented in the patient medical record |
| ESRD | presence of 'ESRD' documented in the medical record |
| Permanent pacemaker | presence of a permanent pacemaker pre-operatively |
| Preop LVEF | LVEF determined by echocardiogram as close to but prior to surgery |
| Preop RV dysfunction | moderate to severe RV impairment as determined by echocardiogram as close, but prior, to surgery |
| MI | documented history of MI in the medical record |
| Arrhythmia | documented history of any rhythm disturbance prior to surgery, either permanent or transient |
| Afib | documented history of atrial fibrillation in the medical record |
| Chronic Lung Disease | based on the documentation of irreversible, symptomatic lung diseases (COPD, asthma, interstitial pneumonia) in the medical record |
| Cerebrovascular disease | documented history of cerebrovascular disease in preoperative notes, or incidence of stroke/TIA/cranial hemorrhage |
| Stroke (table 1) | documented history of stroke (either ischemic or hemorrhagic) in the medical record |
| Peripheral vascular disease | documented history of peripheral vascular disease or history of indicative procedure (peripheral angioplasty, stent, bypass) |
| Endocarditis | presence of endocarditis as primary indication for operation |
| Surgery status | categorized as 'elective' or 'non-elective' based on review of preoperative medical notes |
| Lab values | lab tests closest to but before the operation |
| Procedure coding |  |
| Aorta | patient had aortic graft with or without another concomitant procedure |
| Valve | patient had valve only procedures |
| Valve/CABG | patient had CABG+valve |
| Induction Cardioplegia | initial amount of cardioplegia used to arrest the heart |
| Total calculated crystalloid delivered | calculated crystalloid component of del Nido cardioplegia, based on blood:crystalloid ratio of FD or dilute solution |
| Time to first re-dose | minutes from initial cardioplegia dose to first re-dosing event |
| Re-dose event | cardioplegia delivery greater than 30 minutes from the previous delivery |
| New RV dysfunction, end of case, TEE | moderate to severe RV impairment as determined by intraoperative trans-esophageal echocardiogram at the conclusion of operation, compared to start of operation |
| Postop length of stay | number of days patient remained in the hospital until discharge to home/rehab or death |
| MCS | implementation of ECMO, temporary ventricular assist device, or IABP |
| Stroke (table 3) | based on VARC-2 definition of neurologic impairment with permanent disability or neuroimaging with hemorrhage or infarct |
| Dialysis (table 3) | initiation of renal replacement therapy postoperatively, which was not required preoperatively |
| Arrhythmia | new documented arrhythmia (transient or permanent) in the postoperative period |
| Permanent Pacemaker (table 3) | new implanted permanent pacemaker in the postoperative period |
| Discharge LVEF | LVEF on echocardiogram (TTE or TEE) closest to, but before, hospital discharge or death |
| New discharge RV Dysfunction | moderate to severe RV dysfunction present on echocardiogram (TTE or TEE) closest to, but before, hospital discharge or death - which was not present on preoperative studies |
| ESRD = end stage renal disease; LVEF = left ventricular ejection fraction; RV = right ventricle; MI = myocardial infarction; afib = atrial fibrillation; CABG = coronary artery bypass graft; MCS = mechanical circulatory support | |

Supplementary Table 2 – Missing values from the dataset

| **Field** | **N** | **Remediation** |
| --- | --- | --- |
| Number of re-doses | 2 missing | Patients were excluded from analysis |
| CABG and Other Cases | 4 excluded | Patients were excluded from analysis |
| Hgb | 3 missing | Imputed to the Median |
| WBC | 3 missing | Imputed to the Median |
| Platelets | 3 missing | Imputed to the Median |
| Preop LVEF | 2 missing | Imputed to the Median |
| Preop RV dysfunction | 2 missing | Imputed to the Mode |
| Dialysis | 1 missing | Imputed to the Mode |
| Creatinine | 1 missing | Imputed to the Median |
| Mitral Valve Disease | 1 missing | Imputed to the Mode |
| Mitral Stenosis | 1 missing | Imputed to the Mode |
| Aortic Valve Disease | 1 missing | Imputed to the Mode |
| Aortic Stenosis | 1 missing | Imputed to the Mode |
| Mitral Insufficiency | 1 missing | Imputed to the Mode |
| Tricuspid Insufficiency | 1 missing | Imputed to the Mode |
| Aortic Insufficiency | 1 missing | Imputed to the Mode |
| Hgb = hemoglobin; WBC = white blood cell count | | |

Supplementary Table 3 – Characteristics used for propensity score matching and associated adjusted groups

| **Patient Characteristics** | **Adj. full dose**  **n=48** | **Adj. dilute**  **n=48** | **SMD** |
| --- | --- | --- | --- |
| Age, median [IQR] | 62.4 [51.2-73.7] | 63.2 [56.1-70.5] | 0.037 |
| Male, n(%) | 39 (81.2%) | 39 (81.2%) | <0.001 |
| BMI, median [IQR] | 27.9 [25.4-33.7] | 28.0 [24.9-31.7] | 0.065 |
| Procedure, n(%) |  |  | 0.130 |
| Aorta | 28 (58.3%) | 31 (64.6%) |  |
| Valve | 15 (31.2%) | 13 (27.1%) |  |
| Valve/CABG | 5 (10.4%) | 4 (8.3%) |  |
| Elective Procedure, n(%) | 16 (33.3%) | 15 (31.1%) | 0.045 |
| Diabetes, n(%) | 10 (20.8%) | 11 (22.9%) | 0.050 |
| ESRD, n(%) | 2 (4.2%) | 3 (6.2%) | 0.094 |
| Multiple CCs, n(%) | 9 (18.8%) | 10 (20.8%) | 0.052 |
| Preop LVEF, median [IQR] | 55.0 [50.0- 60.0] | 55.0 [52.5-60.0] | 0.055 |
| Prev Cardiac Surgery, n(%) | 21 (43.8%) | 21 (43.8%) | <0.001 |
| Preop RV Dysfunction, n(%) | 2 (4.2%) | 3 (6.2%) | 0.094 |
| Adj. = Adjusted; SMD = standardized mean difference; BMI = body mass index; ESRD = end stage renal disease; CCs = cross clamps; Preop = preoperative; LVEF = left ventricular ejection fraction; RV = right ventricle | | | |

Supplementary Table 4 - Patient characteristics of entire cohort

| **Patient Characteristics (n=173)** | **N (%), median [IQR]** |
| --- | --- |
| ***Preoperative Info*** | |
| Age (years), median [IQR] | 63.8 [53.9-73.1] |
| Male, n(%) | 115 (66.5%) |
| BMI, median [IQR] | 27.9 [24.8-31.7] |
| ESRD, n(%) | 20 (11.6%) |
| Diabetes, n(%) | 45 (26.0%) |
| CVD, n(%) | 34 (19.7%) |
| Pacemaker, n(%) | 26 (15.0%) |
| PVD, n(%) | 18 (10.4%) |
| Hypertension, n(%) | 131 (75.7%) |
| Lung disease, n(%) | 20 (11.6%) |
| Afib, n(%) | 52 (30.1%) |
| Pre-op LVEF, median [IQR] | 55.0 [50.0-60.0] |
| Pre-op RV Dysfunction, n(%) | 20 (11.6%) |
| Hgb (g/dL), median [IQR] | 12.7 [10.1-14.1] |
| Endocarditis, n(%) | 44 (25.4%) |
| Arrhythmia, n(%) | 67 (38.7%) |
| Immunocompromise, n(%) | 8 (4.6%) |
| CAD, n(%) | 120 (69.4%) |
| PCI, n(%) | 12 (6.9%) |
| Race, White, n(%) | 105 (60.7%) |
| Creatinine (mg/dL), median [IQR] | 1.1 [0.9-1.4] |
| Previous Cardiac Surgery, n(%) | 83 (48.0%) |
| MI, n(%) | 18 (10.4%) |
| Angina, n(%) | 24 (13.9%) |
| Dialysis, n(%) | 12 (6.9%) |
| Stoke, n(%) | 29 (16.8%) |
| Elective Procedure, n(%) | 52 (30.1%) |
| WBC (x10^3^/µL), median [IQR] | 7.7 [6.7-9.4] |
| Platelets (x10^3^/µL), median [IQR] | 207.0 [164.0-259.0] |
| Procedure |  |
| Aorta | 93 (53.8%) |
| Valve | 48 (27.7%) |
| Valve/CABG | 32 (18.5%) |
| Aortic Stenosis, n(%) | 52 (30.1%) |
| Aortic Insufficiency, n(%) | 56 (32.4%) |
| Mitral Stenosis, n(%) | 22 (12.7%) |
| Mitral Insufficiency, n(%) | 69 (39.9%) |
| Tricuspid Insufficiency, n(%) | 45 (26.0%) |
| ***Intraoperative Info*** | |
| Full dose del Nido re-dosing, n(%) | 108 (62.4%) |
| Dilute del Nido re-dosing, n(%) | 65 (37.6%) |
| Cross Clamp Time (min), median [IQR] | 208.0 [189.6-233.4] |
| >1 cross clamp, n(%) | 51 (29.5%) |
| Number of Redoses, n(%) | 3 [2.0-3.0] |
| Total Induction cardioplegia (mL), median [IQR] | 1,070.0 [1000.0-1,330.0] |
| Antegrade, n(%) | 168 (97.1%) |
| Retrograde, n(%) | 71 (41.0%) |
| Time to first re-dose (min), median [IQR] | 73.0 [59.0-91.0] |
| Total re-dose cardioplegia (mL), median [IQR] | 1,110.0 [800.0-1,560.0] |
| Antegrade, n(%) | 148 (85.5%) |
| Retrograde, n(%) | 103 (59.5%) |
| Total cardioplegia (mL), median [IQR]) | 2360.0 [1900.0-2820.0] |
| Total calculated crystalloid delivered (mL), median [IQR] | 1470.0 [1216.0-1920.0] |
| Intraop TEE LVEF (%), start of case, median [IQR] | 55.0 [50.0-55.0] |
| Intraop TEE LVEF (%), end of case, median [IQR] | 50.0 [40.0-55.0] |
| New RV dysfunction, end of case TEE, n(%) | 30 (17.3%) |
| ***Postoperative Events*** | |
| Postop length of stay, median [IQR] | 14.0 [8.0-24.0] |
| New RV dysfunction (at discharge), n(%) | 33 (19.1%) |
| Discharge LVEF, median [IQR] | 55.0 [42.5-58.0] |
| Arrhythmia, n(%) | 114 (65.9%) |
| Pacemaker, n(%) | 34 (19.7%) |
| MI, n(%) | 3 (1.7%) |
| Dialysis, n(%) | 17 (9.8%) |
| Stroke, n(%) | 9 (5.2%) |
| MCS | 38 (22.0%) |
| IABP | 8 (21.1%) |
| VA-ECMO | 17 (44.7%) |
| VAD | 1 (2.6%) |
| IABP/ECMO | 9 (23.7%) |
| IABP/VAD | 1 (2.6%) |
| IABP/ECMO/VAD | 1 (2.6%) |
| ECMO/VAD | 1 (2.6%) |
| In-hospital Mortality, n(%) | 20 (11.6%) |
| BMI = body mass index; ESRD = end stage renal disease; CVD = cerebrovascular disease; PVD = peripheral vascular disease; Afib = atrial fibrillation; LVEF = left ventricular ejection fraction; RV = right ventricle; Hgb = hemoglobin; CAD = coronary artery disease; PCI = percutaneous coronary intervention; MI = myocardial infarction; WBC = white blood cell count; CABG = coronary artery bypass graft; TEE = transesophageal echocardiography; MCS = mechanical circulatory support; IABP = intra-aortic balloon pump; VA-ECMO = veno-arterial extracorporeal membrane oxygenation; VAD = ventricular assist device | |
